# DUSP4 directly deubiquitinates and stabilizes Smad4 protein, promoting proliferation and metastasis of colorectal cancer cells

**DOI:** 10.18632/aging.103823

**Published:** 2020-09-07

**Authors:** Weifeng Xu, Beibei Chen, Dianshan Ke, Xiaobing Chen

**Affiliations:** 1 Department of Medical Oncology, The Affiliated Cancer Hospital of Zhengzhou University, Zhengzhou 450008, Henan, P.R. China; 2 Department of Cell Biology, Southern Medical University, Guangzhou 510515, Guangdong, China

**Keywords:** DUSP4, Smad4, colorectal cancer, EMT, qRT-PCR

## Abstract

Colorectal cancer is a common health-threatening tumor within the gastrointestinal tract. The aim of this study was to test the biological role of DUSP4 in colorectal cancer cells. In our study, DUSP4 overexpression-treated HCT116 cells and DUSP4 knockdown-treated SW480 cells were selected to perform study. Quantitative real-time PCR test (qRT-PCR) and western blot were used to detect DUSP4 abundance in clinical tissues and six cell lines, as well as ubiquitin-related Smad4 degradation. Western blot, migration and invasion. were used to assess the relationships between DUSP4 and Smad4. Higher DUSP4 expression of functional significance was observed in colorectal cancer tissues and cells. The results showed that both treatments could affect the proliferation, colony formation, migration, invasion of tumor cells, and the expression of epithelial mesenchymal transformation (EMT)-associated biomarkers. Moreover, in colorectal cancer cells, DUSP4 could promote the Smad4 degradation by regulating ubiquitin-related Smad4 degradation, and promote the cell proliferation, migration and invasion by regulating Smad4 degradation via Smad4 gene. Meanwhile, DUSP4 can directly deubiquitinate and stabilize Smad4 protein, hence further promote proliferation and metastasis of colorectal cancer cells.

## INTRODUCTION

Colorectal cancer is a common malignant tumor in the gastrointestinal tract [1], of which the symptoms are not obvious in its early stage but worsen as tumor volume increases, and exhibit various forms, such as blood in the stool, diarrhea, local abdominal pain, anemia, and weight loss. Among the digestive system malignancies, its incidence and mortality are second only to gastric, esophageal, and primary liver cancers [2], and the incidence has gradually increased with the changes of people's lifestyles over the decades [3]. Patients with early colorectal cancer have non-specific digestive symptoms, so most of them are in the advanced stage at diagnosis, which triggers a high incidence of postoperative recurrence and metastasis and a poor prognosis [4]. Given the facts above, it’s necessary to demonstrate the molecular mechanisms of colorectal cancer.

Ubiquitination refers to the process of ubiquitin molecule to use a series of special enzymes to sort out and modify target protein molecules in cells [5]. DUSP4, mitogen-activated protein kinase phosphatase 2 (MKP2), is a member of the bispecific phosphatase family and can be inactivated by dephosphorylation of MAPKs [6]. Meanwhile, the protein encoded by DUSP4 belongs to the ubiquitin-specific protease (UBP) family and can cleave ubiquitin from ubiquitinated protein substrates [7], thus making DUSP4 play an important role in the MAPK signaling pathway. Recent studies have shown that DUSP4 protein is highly expressed in breast cancer and liver cancer [8, 9]. The DUSP4 expression is related to tumor cells’ proliferation, apoptosis and metastasis, as well as tumor angiogenesis, for which DUSP4 gene is considered an oncogene [10–13]. However, DUSP4 has not been reported in colorectal cancer. Therefore, the detailed molecular mechanisms of DUSP4 in the occurrence and development of colorectal cancer will provide important clinical references for the early diagnosis and prognosis evaluation of colorectal cancer. Smad4 can directly bind to the E-cadherin gene (CDH1) promoter and inhibit its transcription, and its expression can enhance the tumor cells’ resistance to apoptosis and ability to survive various stress conditions, which leads scholars to recognize it a key protein to control EMT [14–16]. In view of these, the expression of Smad4 in a variety of tumors is related to disease progression, enhanced invasiveness, and poor clinical prognosis [15]. For example, pancreatic cancer cells can undergo Snail 1-mediated EMT process, chemotherapy resistance, and metastasis [17]. In addition, Smad4 is involved in the maintenance of tumor stem cells and inhibition of apoptosis [18]. Therefore, the relationship between Smad4 and tumor invasion and metastasis has inevitably become a hot topic in recent-year study of tumor invasion and metastasis. In colorectal cancer, the mechanism of Smad4 regulation needs further study. Smad4 is an unstable protein that can be tightly linked to multiple E3-ubiquitin ligases. Ubiquitin-modified Smad4 protein can regulate its expression in epithelial cells. Meanwhile, Deubiquitinating enzymes (DUBs) can recognize ligase E3 and remove the ubiquitin molecule (Ub) which is bonded to the target protein and regulates its stability, and the proliferation and metastasis of colorectal cancer cells [19, 20].

In this study, we studied the DUSP4 expression in clinical samples and cell lines. The roles of DUSP4 in cell migration and proliferation were probed. We also demonstrated the detailed molecular mechanisms of DUSP4 molecular in colorectal cancer. The deubiquitinating enzyme DUSP4 was proved to remove the Ub on Smad4 protein.

## RESULTS

### DUSP4 gene was highly expressed in colorectal cancer tissue

**Figure 1:**
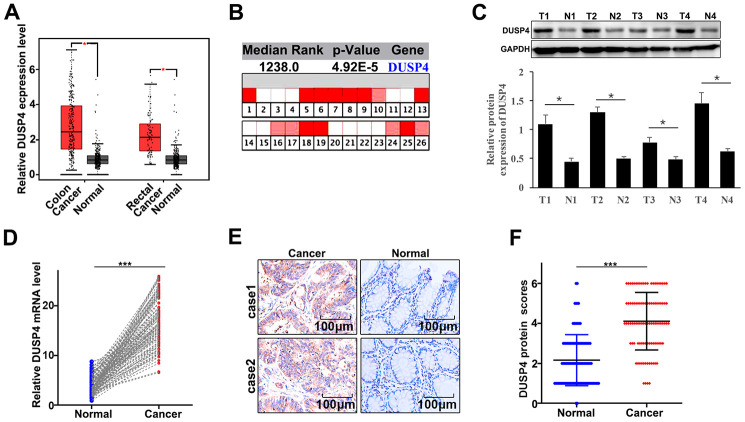
**DUSP4 gene was highly expressed in colorectal cancer tissues.** (**A**) mRNA abundance analysis of DUSP4 gene in GEPIA database. (**B**) mRNA abundance analysis of DUSP4 gene in oncomine. (**C**) Western blot analysis of normal tissues and colorectal cancer tissues. N: normal tissues; T: tumor tissues. GAPDH was employed as an internal reference. (**D**) qRT-PCR analysis of DUSP4 mRNA abundance in four colorectal cancer tissues and the paired normal tissues. (**E** and **F**) Immunohistochemical analysis of normal tissues and colorectal cancer tissues. *P<0.05. ***P<0.001.

Firstly, we detected the expression of DUSP4 in colorectal cancer tissues and the paired normal tissues through online dataset, western blot, qRT-PCR, and immunohistochemical staining analysis. For online dataset analysis, GEPIA database (http://gepia.cancer-pku.cn/index.html) and Oncomine database (https://www.oncomine.org/) were adopted for evaluation, and it was found that DUSP4 expression was higher in colorectal cancer tissues than in the paired normal tissues (P<0.05) (Figure 1A and 1B). Meanwhile, western blot and qRT-PCR, as well as photochemical staining analyses both indicated that DUSP4 expression was significantly higher in colorectal cancer tissues than in the paired normal tissues (Figure 1C–1F). In summary, DUSP4 expression was higher in colorectal cancer tissues than in the paired normal tissues.

### DUSP4 promoted metastasis and proliferation of colorectal cancer cells *in vitro*

**Figure 2:**
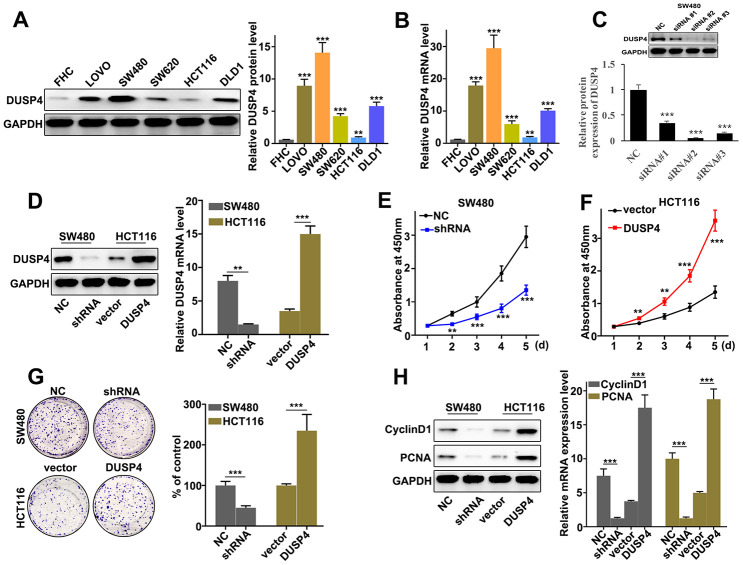
**DUSP4 promoted metastasis and proliferation of colorectal cancer cells *in vitro.*** (**A**) Western blot analysis of DUSP4 expression in FHC, LOVO, SW480, SW620, HCT116, and DLD1. (**B**) qRT-PCR analysis of DUSP4 expression in FHC, LOVO, SW480, SW620, HCT116, and DLD1. (**C**) Knockdown treatment of three designed siRNAs in SW480 cells. (**D**) DUSP4 protein expression of DUSP4 knockdown-treated SW480 cells and DUSP4 overexpression-treated HCT116 cells. (**E**) Cell proliferation analysis of DUSP4 knockdown-treated SW480 cells. (**F**) Cell proliferation analysis of DUSP4 overexpression-treated HCT116 cells. (**G**) Colony formation analysis of DUSP4 knockdown-treated SW480 cells and DUSP4 overexpression-treated HCT116 cells. (**H**) Western blot analysis of cell proliferation-related biomarkers expression in DUSP4 knockdown-treated SW480 cells and DUSP4 overexpression-treated HCT116 cells. **P<0.01, ***P<0.001.

In order to further probe the biological role of DUSP4 in colorectal cancer cells, DUSP4 expression was assessed in six selected cell lines, namely FHC, LOVO, SW480, SW620, HCT116, and DLD1. Western blot and qRT-PCR analysis indicated that DUSP4 protein expression was significantly different between each line (Figure 2A and 2B). It was notable that DUSP4 protein expression was at the lowest level in HCT116 cells (P<0.01), and at the highest level in SW480 cells (P<0.001). Therefore, HCT116 and SW480 cells were selected into next study. The two kinds of cells underwent DUSP4 over-expressed and knocked-down, respectively. Figure 2C showed that siRNA #3 was an effective molecular for DUSP4 knockdown protein expression in SW480 cells, so the siRNA # 3 treated SW480 cells were used to do analysis. Meanwhile, western blot analysis was employed to determine the DUSP4 protein expression in HCT116 and SW480 cells under different treatments. Figure 2D suggested a success respectively in the overexpression and knockdown of DUSP4 in HCT116 and SW480 cells. In term of cell proliferation analysis, DUSP4 knockdown could significantly reduce cell proliferation in SW480 cells compared to normal SW480 cells on day 2, 3, 4, 5 (Figure 2E) (P<0.01). However, DUSP4 overexpression could significantly promote cell proliferation in HCT116 cells compared to vector-treated HCT116 cells on day 2, 3, 4, 5 (Figure 2F) (P<0.01). Moreover, cell colony numbers were significantly increased in DUSP4 over-expressed HCT116 cells compared to vector-treated HCT116 cells (Figure 2G) (P<0.01), but were significantly decreased in DUSP4 knocked-down SW480 cells compared to normal SW480 cells (Figure 2G) (P<0.01). Meanwhile, cell proliferation-related biomarkers such as CyclinD1 and PCNA in DUSP4 knocked-down SW480 cells and DUSP4 over-expressed HCT116 cells were detected by western blot and qRT-PCR. The results suggested that the expression of CyclinD1 and PCNA was lower in DUSP4 knocked-down SW480 cells than in normal SW480 cells (P<0.05) (Figure 2H), but was higher in DUSP4 over-expressed HCT116 cells than in normal HCT116 cells (P<0.05) (Figure 2H). In summary, *in vitro,* DUSP4 overexpression in HCT116 cells could promote metastasis and proliferation of colorectal cancer cells whereas DUSP4 knockdown in SW480 cells could restrain cell metastasis and proliferation.

### Regulation of DUSP4 on colorectal cancer cell migration and invasion

**Figure 3:**
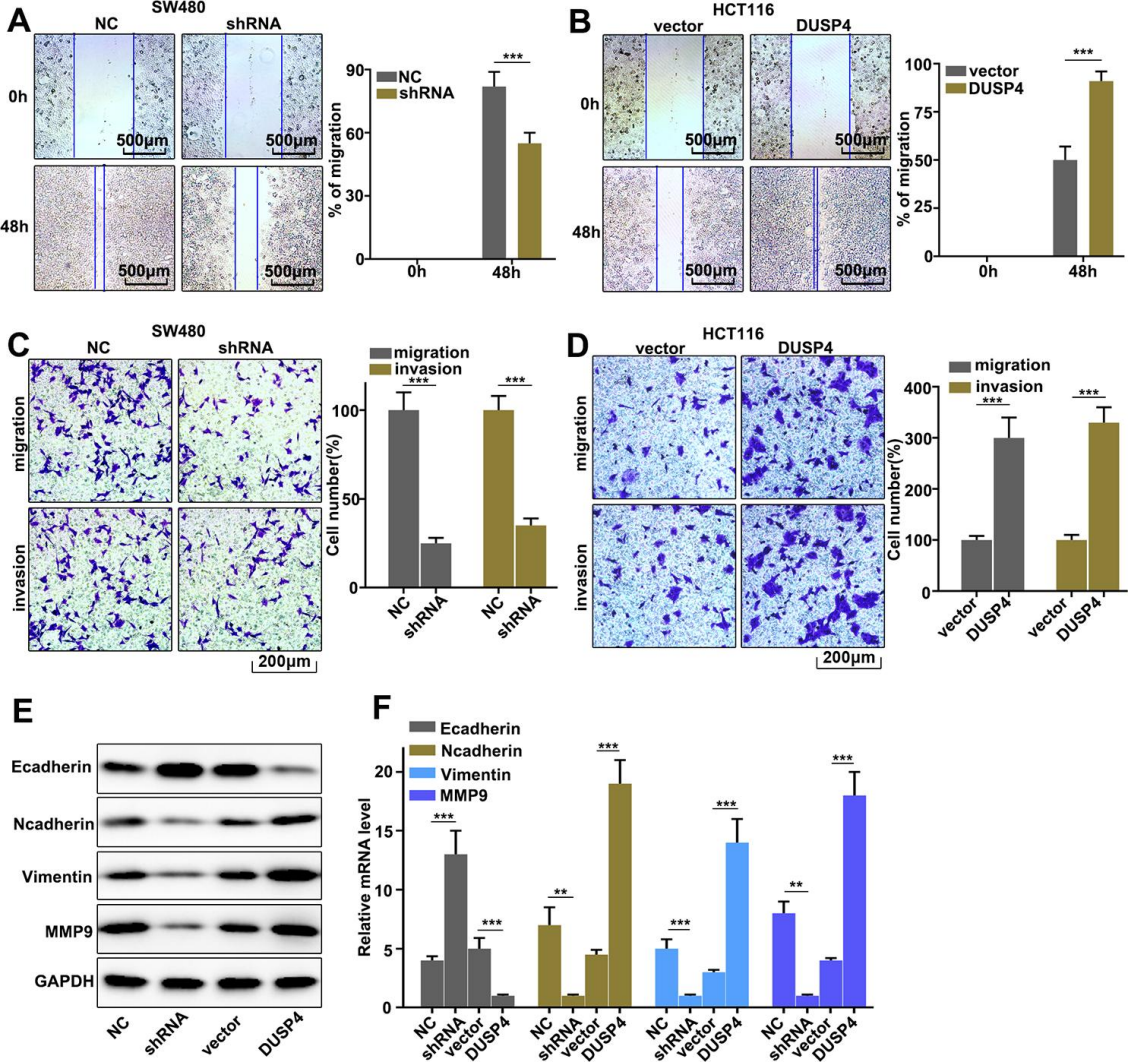
**Regulation of USP4 on colorectal cancer cell migration and invasion.** (**A**) Cell scratch test of DUSP4 knockdown-treated SW480 cells. (**B**) Cell scratch test of DUSP4 overexpression-treated HCT116 cells. (**C** and **D**) Cell migration and invasion analysis of DUSP4 knockdown-treated SW480 cells and DUSP4 overexpression-treated HCT116 cells, respectively. (**E**) Western blot analysis of EMT-related biomarkers expression in DUSP4 knockdown-treated SW480 cells and DUSP4 overexpression-treated HCT116 cells. (**F**) qRT-PCR analysis of EMT-related biomarkers expression in DUSP4 knockdown-treated SW480 cells and DUSP4 overexpression-treated HCT116 cells. **P<0.01, ***P<0.001.

Our work discussed the role of DUSP4 in regulating colorectal cancer cell migration and invasion in DUSP4 over-expressed HCT116 cells and DUSP4 knocked-down SW480 cells. The results showed that DUSP4 knockdown in SW480 cells could significantly inhibit cell migration compared to normal SW480 cells (Figure 3A) (P<0.01), whereas DUSP4 overexpression in HCT116 cells could significantly promote cell migration compared to normal HCT116 cells (Figure 3B) (P<0.01). Moreover, cell migration and invasion in DUSP4 over-expressed HCT116 cells and DUSP4 knockdown SW480 cells were further studies, and it was found that DUSP4 knockdown in SW480 cells could significantly inhibit cell migration and invasion compared to normal SW480 cells (Figure 3C) (P<0.01), but DUSP4 overexpression in HCT116 cells could promote cell migration and invasion compared to normal HCT116 cells (Figure 3D) (P<0.01). In addition, we further analysed the protein expression of E-cadherin, N-cadherin, Vimentin, and MMP9, and found that DUSP4 knockdown in SW480 cells could effectively inhibit protein expression of N-cadherin, Vimentin, and MMP9, and that DUSP4 overexpression in HCT116 cells could effectively increase protein expression of N-cadherin, Vimentin, and MMP9 (Figure 3E and 3F) (P<0.01). Additionally, protein expression of E-cadherin was effectively promoted by DUSP4 knockdown in SW480 cells (P<0.01) but inhibited by DUSP4 overexpression in HCT116 cells(P<0.01). Therefore, DUSP4 overexpression in HCT116 cells could promote the protein expressions of N-cadherin, MMP9, and Vimentin, but inhibit E-cadherin. Meanwhile, DUSP4 knockdown in SW480 cells could inhibit the protein expressions of N-cadherin, MMP9, and Vimentin, but promote E-cadherin.

### DUSP4 down-regulated Smad4 expression

**Figure 4:**
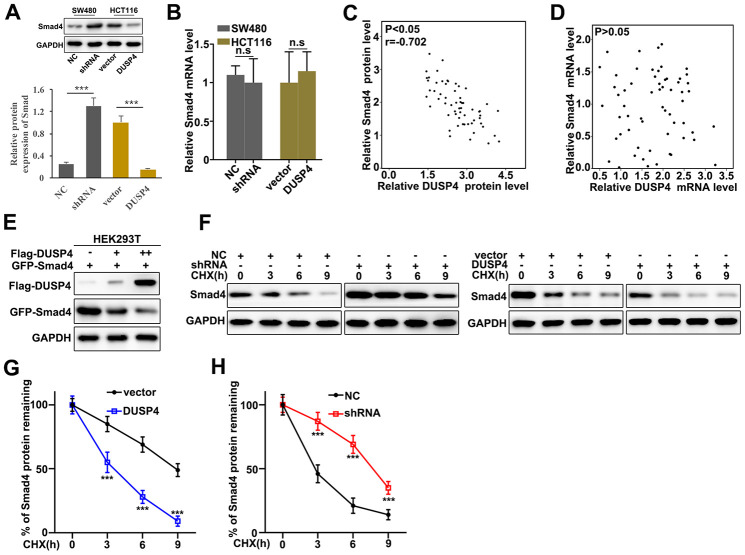
**DUSP4 down-regulated Smad4 gene expression.** (**A**) Western blot analysis of Smad4 protein expression in DUSP4 knockdown-treated SW480 cells and DUSP4 overexpression-treated HCT116 cells. (**B**) qRT-PCR analysis of Smad4 mRNA expression in DUSP4 knockdown-treated SW480 cells and DUSP4 overexpression-treated HCT116 cells. (**C**) Correlation analysis of DUSP4 protein and Smad4 protein in clinical samples. (**D**) Correlation analysis of DUSP4 mRNA and Smad4 mRNA in clinical samples. (**E**) Relationship analysis of DUSP4 and Smad4 in HEK293T cells which was transfected with DUSP4 and Smad4. (**F**–**H**) Relative Smad4 protein expressions in DUSP4 knockdown-treated SW480 cells and DUSP4 overexpression-treated HCT116 cells with cyclohexane (CHX) treatment at different time points. **P<0.01, ***P<0.001.

Potential relationships between the expressions of DUSP4 and Smad4 was assessed. Western blot and qRT-PCR were employed to investigate the protein and mRNA expressions in DUSP4 over-expressed HCT116 cells and DUSP4 knocked-down SW480 cells. Figure 4A showed that Smad4 expression was higher in DUSP4 knocked-down SW480 cells than in normal SW480 cells, but was less abundant in over-expressed HCT116 cells than in normal HCT116. It was notable that no difference of Smad4 mRNA abundance was detected in DUSP4 over-expressed HCT116 cells and DUSP4 knocked-down SW480 cells (Figure 4B). The above results suggested that DUSP4 could affect Smad4 protein expression but not Smad4 mRNA abundance. We also further analyzed the potential relationships of the mRNA and protein expressions of DUSP4 and Smad4 in clinical samples (Figure 4C and 4D). The results suggested a possible correlation between the protein expressions of DUSP4 and Smad4 (Figure 4C), but no correlation between the mRNA expressions of DUSP4 and Smad4 (Figure 4D). Based on the facts above, we employed DUSP4 and Smad4 to transfect HEK293T cells to probe the potential relationships between protein expressions of DUSP4 and Smad4. Figure 4E showed the negative correlation between protein expressions of DUSP4 and Smad4. Cycloheximide (CHX), which can inhibit protein synthesis, was also employed to study the relationships between protein expressions of DUSP4 and Smad4 in DUSP4 over-expressed HCT116 cells and DUSP4 knocked-down SW480 cells. Figure 4F–4H showed that DUSP4 overexpression in HCT116 cells could significantly reduce the remaining Smad4 protein in cells compared to normal HCT116 cells (Figure 4F and 4G) (P<0.01), and DUSP4 knocked-down SW480 cells could significantly promote the remaining Smad4 protein in cells compared to normal SW480 cells (Figure 4F and 4H) (P<0.01). In summary, DUSP4 protein expression was negatively related to Smad4 expression.

### DUSP4 regulated Smad4 through ubiquitination

**Figure 5:**
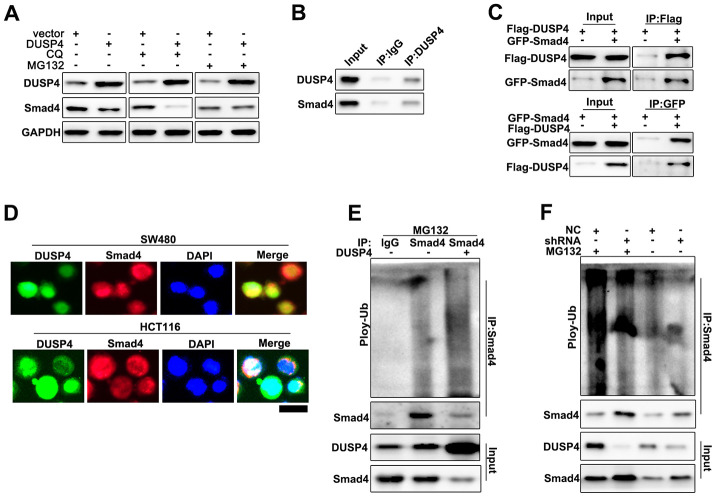
**DUSP4 regulated Smad4 expression through ubiquitination.** (**A**) Western blot analysis of DUSP4 and Smad4 expression in DUSP4 overexpression-treated HCT116 cells with CQ and MG132 treatment at different time points. (**B**) CO-IP analysis of DUSP4 and Smad in SW480 cells. (**C**) Forward and reverse CO-IP analysis of DUSP4 and Smad in SW480 cells and HCT116 cells. (**D**) Immunocolocalization analysis of DUSP4 and Smad in SW480 cells and HCT116 cells. (**E**) Ubiquitination test of DUSP4 and Smad in HCT116 cells. (**F**) Ubiquitination test of DUSP4 and Smad in SW480 cells. Scale bar: 5 μm.

Potential relationships between DUSP4 ubiquitination and Smad4 in DUSP4 over-expressed HCT116 cells was evaluated. MG132 is an intracellular inhibitor of the proteasome degradation pathway, and Meanwhile, Chloroquine (CQ) is an inhibitor of the autophagolysosomal degradation pathway. Therefore, the two molecules were employed to study the protein expressions of DUSP4 and Smad4 in DUSP4 over-expressed HCT116 cells (Figure 5A). The results suggested that MG132 could effectively reduce Smad4 protein expression whereas CQ had no effect on Smad4 protein expression. Therefore, we speculated that DUSP4 could regulate Smad4 expression through the ubiquitination but not the cellular autophagy pathway. Furthermore, we examined the potential interaction between DUSP4 protein and Smad4 protein in cell, and Co-immunoprecipitation (Co-IP) was used Figure 5B showed that DUSP4 protein could interact with Smad4 protein. The forward and reverse protein Co-IP of DUSP4 protein and Smad4 protein in DUSP4 over-expressed HCT116 cells was carried out and the interaction between DUSP4 protein and Smad4 protein was further identified (Figure 5C). Immunofluorescence colocalization analysis of DUSP4 protein and Smad4 protein revealed that the spatial distribution of DUSP4 and Smad4 was overlapping. The result above further illustrated a mutual combination between DUSP4 and Smad4 in DUSP4 over-expressed HCT116 cells (Figure 5D). In addition, we carried out ubiquitination test in DUSP4 over-expressed HCT116 cells and DUSP4 knocked-down SW480 cells. It was showed that Smad4 ubiquitination degradation was promoted by the overexpression of DUSP4 in HCT116 cell line (Figure 5E), but was inhibited by the knockdown of DUSP4 in SW480 cells (Figure 5F). In summary, DUSP4 could regulate Smad4 gene expression through ubiquitination.

### Rescue experiment

**Figure 6:**
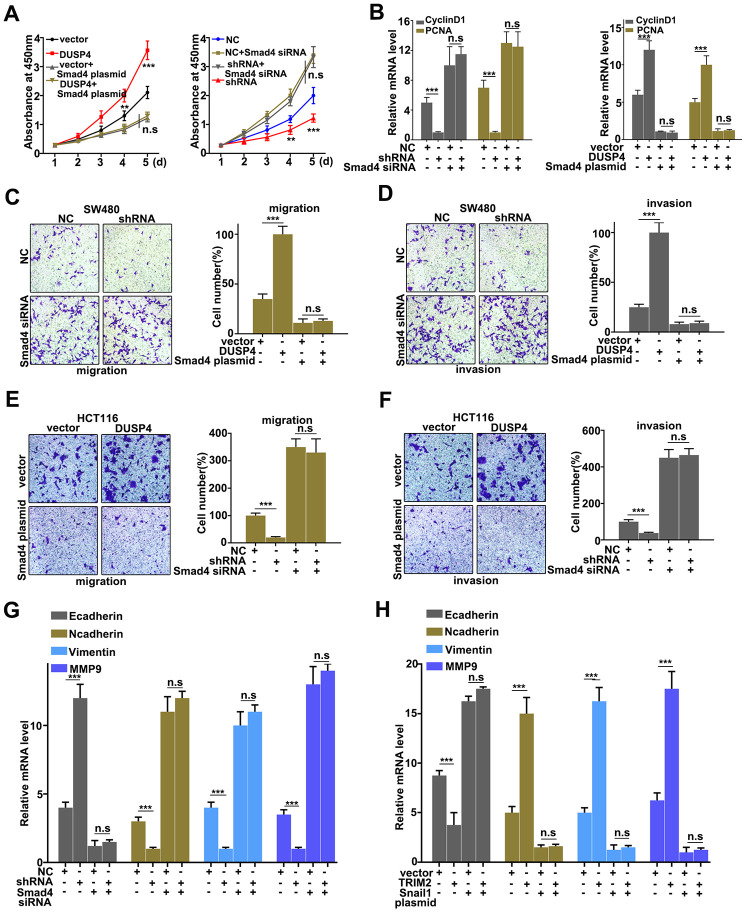
**Rescue experiment.** (**A**) CCK8 analysis of DUSP4 knockdown-treated SW480 cells and DUSP4 overexpression-treated HCT116 cells that had been treated with Smad 4 knockdown and overexpression treatments. (**B**) Western blot and qRT-PCR analysis of Cyclin D and PCNA expression in USP4 knockdown-treated SW480 cells and DUSP4 overexpression-treated HCT116 cells that had been treated with Smad 4 knockdown and overexpression treatments. (**C** and **D**) Migration and invasion analysis of DUSP4 knockdown-treated SW480 cells that had been treated with Smad4 overexpression treatment. (**E** and **F**) Migration and invasion analysis of DUSP4 overexpression-treated HCT116 cells that had been treated with Smad 4 knockdown treatment. (**G** and **H**) qRT-PCR analysis of EMT related biomarkers in DUSP4 knockdown-treated SW480 cells and DUSP4 overexpression-treated HCT116 cells that had been treated with Smad 4 knockdown and overexpression treatments. **P<0.01, ***P<0.001.

Rescue experiment was performed to demonstrate the relationships between DUSP4 and Smad4. We further introduced Smad4 knockdown and overexpression treatment respectively in DUSP4 over-expressed HCT116 cells and DUSP4 knocked-down SW480 cells. Figure 6A showed that Smad4 knockdown and overexpression treatment could effectively promote and inhibit cell proliferation changes caused by DUSP4 overexpression in HCT116 cells and DUSP4 knockdown in SW480 cells, respectively (Figure 6A). We also further examined the mRNA expressions of Cyclin D1 and PCNA, biomarkers for cell proliferation, in Smad4 knockdown and overexpression treated HCT116 cells and SW480 cells. Figure 6B suggested that Smad4 knockdown and overexpression treatment could effectively promote and inhibit the expressions of Cyclin D1 and PCNA changes caused by DUSP4 overexpression in HCT116 cells and DUSP4 knockdown in SW480 cells, respectively. Furthermore, we studied the cell migration and invasion of the Smad4 knocked-down and over-expressed HCT116 cells and SW480 cells, which had been treated with DUSP4 overexpression and DUSP4 knockdown, respectively. Figure 6C and 6D showed that Smad4 overexpression-treated SW480 cells could effectively reverse the changes of migration and invasion caused by DUSP4 knockdown in SW480 cells (P<0.01). Meanwhile, Figure 6E and 6F showed that Smad4 knockdown-treated HCT116 cells could effectively reverse the changes in migration and invasion caused by DUSP4 overexpression in HCT116 cells (P<0.01). In addition, protein expressions of E-caderin, N-caderin, Vimentin, and MMP9, which were all the biomarkers of EMT, were detected in differently-treated groups. Results showed that Smad4 knockdown-treated HCT116 cells could effectively reverse the protein changes of EMT-related biomarkers caused by DUSP4 overexpression in HCT116 cells (Figure 6G) (P<0.01), while Smad4 overexpression-treated SW480 cells could effectively reverse the protein changes of EMT-related biomarkers caused by DUSP4 knockdown in SW480 cells (Figure 6H) (P<0.01). In summary, knockdown and overexpression of Smad4 could effectively rescue the changes of cell proliferation, migration, invasion, and EMT in DUSP4 over-expressed and knocked-down HCT116 cells and SW480 cells.

## DISCUSSION

Ubiquitination is an imperative part in numerous biological processes such as cellular localization, protein degradation and metabolism, which takes part within the control of nearly all life activities, including cell cycle, proliferation, apoptosis, differentiation, metastasis, gene expression, transcription regulation, signal transmission, damage repairment, inflammatory immunity and so on [21]. Previous studies suggested that ubiquitination was closely related to the onset of diseases such as tumors and cardiovascular disease [22, 23]. DUBs recognize ligase E3 and remove the Ub bound to the target protein, thereby reversing the ubiquitination process [24]. There have been reported a total of 98 deubiquitinating, which were mainly divided into four categories, namely cysteine protease (Ub-specific protease (USP)), Ub carboxyl-terminal hydrolase (UCH), ovarian tumor-like protease (OTU), and zinc metalloenzymes [25]. In primary breast cancer, MKP-2 / DUSP4 is expressed several times higher than normal tissues. Study has shown that tamoxifen can increase MKP-2 expression in breast cancer. MKP-2 overexpression can reduce cell proliferation and increase the cells' sensitivity to tamoxifen through ERK [26]. In neoadjuvant chemotherapy, over-expressed DUSP4 can increase chemotherapy-induced apoptosis, and knocked-down DUSP4 can activate the Ras-ERK signaling pathway in BLBC [27]. In addition, DUSP4 is a negative regulator of ERK. DUSP4 down-expression could promote the proliferation and survival of TNBC tumor cells [28]. In this study, we identified that DUSP4 expression was significantly higher in colorectal cancer tissues and cells than in normal tissues and cells. We also studied the biological role of DUSP4 in DUSP4 over-expressed HCT116 cells and DUSP4 knocked-down SW480 cells. The results indicated that DUSP4 could regulate the metastasis and proliferation of colorectal cancer cells *in vitro*. In addition, DUSP4 was closely related to the regulation of colorectal cancer cell migration and invasion. Meanwhile, DUSP4-related ubiquitin signaling pathway play a core role in colorectal cancer cell function. The above results suggested DUSP4 was an important molecular to the function and behaviors of colorectal cancer cell.

Tumor metastasis is a major factor that threatens the survival of cancer patients [29]. In previous studies, low E-Cadherin expression was thought to be closely related to tumor metastasis, which makes EMT defined by a decreased expression of the epithelial connexin E-cadherin [30, 31]. One important characteristic of EMT is that the expression of epithelial marker proteins such as E-cadherin is down-regulated and the expression of mesenchymal marker proteins such as vimentin is up-regulated [30]. EMT has been identified to be present in multiple epithelial-derived malignancies [32]. In this study, it was notable that Smad4 (EMT biomarker) expression was significantly affected in DUSP4 over-expressed HCT116 cells and DUSP4 knocked-down SW480 cells. Smad4 can directly interact with DUSP4 in cell and DUSP4 can reduce Smad4 protein expression without affecting its transcription. Protein stability is mainly affected by proteasome degradation pathways and autophagolysosomal degradation pathways [33]. Our study suggested that DUSP4 did affect the protein degradation pathway of Smad4 through ubiquitination modification. Meanwhile, DUSP4 over-expressed HCT116 cells and DUSP4 knocked-down SW480 cells with knockdown and overexpression of Smad4 could affect cell migration, invasion, and EMT-related biomarkers such as E-caderin, N-caderin, Vitmentin, and MMP9. It had been proved that E-cadherin was down-regulated and mesenchymal marker proteins such as vimentin was up-regulated in EMT [30]. Our results revealed that DUSP4 and Smad4 could regulate EMT of colorectal cancer through E-caderin, N-caderin, Vitmentin, and MMP9 gene.

## CONCLUSION

In summary, we demonstrated that high DUSP4 expression could be detected in colorectal cancer tissues and cells. DUSP4 could aggravate cell proliferation, invasion, and migration in colorectal cancer by regulating Smad4 ubiquitylation degradation. Therefore, DUSP4 plays a biological role in tumor promotion, but Smad4 might act a suppressive effect. Our results could provide detailed information for further study in colorectal cancer.

## MATERIALS AND METHODS

### Tissue samples and cells

Four colorectal cancer samples and their paired normal tissues were collected in the Department of pathology, the Affiliated Cancer Hospital of Zhengzhou University between March 2018 and May 2019. The information of four patients is listed as follows: gender: male, average age: 62±3.4, stage: T3. All experimental protocols were reviewed and approved by the ethics committee of the Affiliated Cancer Hospital of Zhengzhou University. All patients had read and signed the informed consent. The collected tissues were quickly solidified within the fluid nitrogen and stored at -80°C until further study. FHC, LOVO, SW480, SW620, HCT116, and DLD1 cells were purchased from ATCC (Virginia, USA). Cells were cultured with RPMI 1640 with 10% (v/v) FBS (Invitrogen, Carlsbad, CA) in a humidified chamber at 5% CO_2_, at 37°C. HCT116 Cells were planted into six-well plates (5×10^5^ cells per well). DMEM with 10% FBS without penicillin and streptomycin was used as culture medium. OPTI-MEM serum-free medium (M5650, Sigma Aldrich) and Lipofectamine 2000 reagent (Thermo Fisher Scientific, USA) were used in transfection tests. Final concentration of 100 nM siRNA was introduced in this study. Meanwhile, pEZ-Lv201 Vector was employed to construct the DUSP4 overexpression system in SW480 cells, and used as the negative control in normal SW480 cells. Lentiviral particles generated with a standardized protocol were used to produce the highly purified plasmids. Endo Fectin-Lenti^TM^ and Titer Boost^TM^ reagents (FulenGen, Guangzhou, China) were used to co-transfect SW480 cells. The supernatant was collected after 48-hour transfection and stored at -80°C.

### qRT-PCR analysis

Total RNA was extracted with TRNzol Universal RNA Extraction Kit (TIANGEN BIOTECH(BEIJING)CO, LTD). The mRNA expression was detected with Bio-Rad IQ5 system. The PCR reaction contained 10μL GoldStar Probe Mixture (Low ROX) (CWBio, China), 1μL sense primer (10 nM), 1μL anti-sense primer (10 nM), 2μL cDNA template (10 ng), and 6μL H_2_O. The qRT-PCR program was set as following: 95°C, 30 seconds, 40 cycles (95°C, 5 seconds, and 60°C, 10 seconds). 2-ΔΔCt cycle method was used to calculate the relative expression level of mRNAs. GAPDH was employed as the internal control. Primer sequences were listed as follows: DUSP4: Forward Primer_GGCGGCTATGAGAGGTTTTCC, Reverse Primer_TGGTCGTGTAGTGGGGTCC; GAPDH: Forward Primer_TGTGGGCATCAATGGATTTGG, Reverse Primer_ACACCATGTATTCCGGGTCAAT; Smad4: Forward Primer_CTCATGTGATCTATGCCCGTC, Reverse Primer_AGGTGATACAACTCGTTCGTAGT; MMP9: Forward Primer_TGTACCGCTATGGTTACACTCG, Reverse Primer_GGCAGGGACAGTTGCTTCT; E-Cadherin: Forward Primer_CGAGAGCTACACGTTCACGG, Reverse Primer_GGGTGTCGAGGGAAAAATAGG; N-Cadherin: Forward Primer_TTTGATGGAGGTCTCCTAACACC, Reverse Primer_ACGTTTAACACGTTGGAAATGTG; Vimentin: Forward Primer_AGGCAAAGCAGGAGTCCACTGA, Reverse Primer_ATCTGGCGTTCCAGGGACTCAT; Cyclin D1: Forward Primer_GCTGCGAAGTGGAAACCATC, Reverse Primer_CCTCCTTCTGCACACATTTGAA; PCNA: Forward Primer_CCTGCTGGGATATTAGCTCCA, Reverse Primer_CAGCGGTAGGTGTCGAAGC.

### Western blot analysis

Cellular protein in three distinctive groups was lyzed by 1% PMSF an RIPA lysis buffer (89900, ThermolFisher Scientific). The total protein was reacted with SDS-PAGE, and further examined by sodium dodecy lsulfate–polyacrylamide gel electrophoresis. Then, the proteins were transferred onto a polyvinylidene difluoride layer (EI9051, ThermolFisher Scientific). After being blocked for 1 hour at room temperature, the layer was brooded with anti-Rabbit DUSP4 (1:1000) (#5149, CST, USA), GAPDH (1:1000) (#2118, CST, USA), Cyclin D1 (1:1000) (#2978 CST, USA), PCNA (1:1000) (#13110 CST, USA), E-Cadherin (1:1000) (#31958, CST, USA), N-Cadherin (1:1000) (#13116, CST, USA), Vimentin (1:1000) (#5741, CST, USA), MMP9 (1:1000) (#13667, CST, USA), and Smad4 (1:1000) (#46535, CST, USA) overnight. Proteins were hatched with secondary antibodies for 1 hour at room temperature. After being treated with ECL Chemiluminescence Detection Kit (PromoCell, German), the bands were observed with Chemiluminescence Imaging (clinx Ltd., China).

### Immunohistochemical staining analysis

The immunohistochemical SP method was used to stain cancer tissue sections. Tissue sections were first cultured in a 60 °C incubator for 60 minutes and then subjected to multiple treatments, including immersion in xylene to dewax, gradient alcohol hydration, microwave antigen repair, and 3% hydrogen peroxide treatment. After blocking the goat serum, the sections were added into an anti-rabbit DUSP4 monoclonal antibody (1: 600) (#5182, CST, USA) and incubated at 4°C overnight. An optical microscope was used for observation.

### Migration and invasion assay

Oris Cell Migration Assay Kit (Platypus, USA) and EZCell Cell Invasion Assay Kit (Biovision, USA) were used to perform cell migration and cell invasion assays, respectively. The detailed steps were strictly followed in accordance with the instruction provided by the manufacturer.

### MTT

The treated cell suspension with a density of 4,000 cells/well was seeded into 96-well plates. The cells in each group were cultured in 5% CO_2_/37 °C environment. Proliferation ability of the 4-group cells was detected at the first, second, third, fourth, and fifth day after treatments, respectively. MTT kit was obtained from Sigma-Aldrich (USA). A 20 μl of MTT solution was added to each well, and cells were cultured for 4 hours. The culture medium was carefully removed into each well. A 150 ul of dimethyl sulfoxide was added in per well. The absorbance at OD450 was measured after the crystals were thoroughly dissolved. Then, cell proliferation was calculated.

### Colony formation

Cells were seeded into a 6-well plate with a density of 1000 cells/well, and cultured in 37 °C/5% CO_2_. The cell clone size was observed, and the medium was changed according to the medium condition. When macroscopic clones appeared, the culture was terminated. The medium in the well was discarded. The well was washed twice with PBS, and air-dried. Cells were then fixed with 4% paraformaldehyde for 30 minutes. After drying, it was stained with 1% crystal violet dye solution for 30 minutes. Subsequently, cell colony formation was observed under an optical microscope.

### Immunofluorescence analysis

Colon cancer cells in the logarithmic growth phase were inoculated into 24-well plates with cell slides and cultured for 48 hours. We discarded the medium, removed the cell slides, and washed 3 times with PBS. Sections were fixed with 4% paraformaldehyde at 4° C for 30 minutes and then washed 3 times at room temperature with PBS for 5 minutes. Furthermore, 0.1% Triton was used to treat sections for 10 minutes and PBS was used to wash sections for 5 minutes. The goat serum incubation section was blocked for 1 hour at room temperature. Subsequently secondary antibody was used to react for 1 hour at room temperature. After washing 3 times with PBS for 10 minutes each time, an inverted fluorescence microscope was used to observe the results.

### Co-IP detection

Colon cancer cells in logarithmic growth phase was adopted for detection. Total protein was extracted by using RIPA Lysis and Extraction Buffer (89900, ThermolFisher Scientific, USA). In short, we washed the beads with a 100 μL of ice-cold buffer. A 100 μL of antibody binding buffer was added to spin the antibody and magnetic beads for 30 min. Then the beads were washed 3 times with 200 μL buffer for 5 minutes each time. Cell lysate and antibody-conjugated magnetic beads were incubated for 1 hour at room temperature and washed 3 times with 200 μL buffer for 5 minutes each time. A 20 μL of elution buffer was used to wash the beads once and the supernatant was taken.

### Statistical methods

In this study, we have employed Graphpad 5. 0 software to analysis data. Student’s t-test was used for comparison of the groups. P values < 0.05 were considered statistically significant.
